# Effect of processing techniques on energy content and amino acid digestibility in corn germ meal fed to growing pigs

**DOI:** 10.5713/ab.24.0581

**Published:** 2025-02-27

**Authors:** Jinbiao Zhao, Zhaoyu Liu, Xiaoming Song, Meiyu Yang, Zhongchao Li, Ling Liu

**Affiliations:** 1 State Key Laboratory of Animal Nutrition and Feeding, College of Animal Science and Technology, China Agricultural University, Beijing, China

**Keywords:** Amino Acids, Corn Germ Meal, Energy Content, Growing Pigs, Processing Technology

## Abstract

**Objective:**

The study was conducted to determine energy contents, apparent total tract digestibility (ATTD) of nutrients, the apparent (AID) and standardized (SID) ileal digestibility of amino acids (AA) in corn germ meals (CGM) produced by processing technologies of wet milling (CGM-CV1 and CGM-CV2), heating-dried CGM with steep liquor (CGMSL-DH) and dried using indirect heat (CGMSL-IH), corn germ expellers (CGE) and dry-grind processing method (CGM-DG).

**Methods:**

In Exp. 1, forty-two crossbred male barrows with an initial body weight (BW) of 51.2±4.5 kg were assigned to 1 of 7 diets in a randomized complete block design. In Exp. 2, seven cannulating barrows with an initial BW of 35.3±1.3 kg were assigned to 7 diets in each period according to a Latin square design. Each diet included 6 replicates.

**Results:**

The ATTD of acid detergent fiber and gross energy (GE), and SID of most AA, except for tryptophan (Trp) and cysteine (Cys), in CGMSL-IH was greater (p<0.05) than those in CGM-CV2. The ATTD of organic matter (OM), crude protein and GE, and SID of most AA, except for arginine (Arg), histidine (His), lysine (Lys) and tyrosine (Tyr), in CGMSL-DH was greater (p<0.05) than those in CGM-CV1 for pigs. The ATTD of GE and acid-hydrolyzed ether extract, contents of digestible and metabolizable energy (ME), and SID of AA, except for Arg, His, Lys, Phenylalanine, Trp, Cys, glutamic acid, glycine and Tyr, in CGE were greater (p<0.05) than those in CGM-CV1. The ATTD of OM and GE, and SID of all AA in CGM-DG were greater (p<0.05) than those in CGM-CV1. Compared with the CGM-CV1, CGM-CV2 showed a greater (p<0.05) ATTD of GE and SID of some AA for growing pigs.

**Conclusion:**

Different processing technologies lead to large variations in energy contents and AA digestibility of CGM for growing pigs. The CGE contains more available energy compared with CBM-CV1, and CGMSL-DH, CGMSL-IH and CGM-DG provides more ileal digestibility of AA for growing pigs compared with the CGM-CV1 and CGM-CV2.

## INTRODUCTION

Corn germ meal (CGM) as one of cereal processing by-products is commonly used to provide energy and protein in the diets for pigs and poultry. Previous studies reported that the contents of digestible energy (DE) and metabolizable energy (ME) in CGM for growing pigs ranged from 14.20 MJ/kg dry matter (DM) to 15.66 MJ/kg DM and from 12.99 MJ/kg DM to 14.64 MJ/kg DM [1,2], respectively. In practice, CGM is the residue remaining after oil extraction of the corn germ, and different oil extraction technologies would lead to large differences in the chemical composition and energy contents. Importantly, anti-nutritional factors, such as phytic acid and fiber components, hinder dietary nutrients digestibility and decrease growth performance of pigs. As a result, CGM has been included at levels up to 30% in the diet without disturbing economical parameters for growing-finishing pigs [3]. However, the nutrients profiles of CGM derived from different sources are highly variable due to different processing conditions. Processing technologies included traditional solvent extraction [4], the quick germ-quick fiber techniques [5] and expeller pressing [6], respectively. A previous report showed that the prefractionation process of quick germ-quick fiber was an engineering economic model for production of corn by-products with the balanced protein and fiber contents compared with the conventional corn dry-grind process [7]. Corn steep liquor (SL) has been traditionally dealt with by combining it with corn fiber to produce corn gluten feed [8]. In recent years, the CGM is always mixed with the corn SL, and then the blended material is dried via direct heat or indirect heat to prepare the feed ingredients for diet formulation [9]. CGM is produced by the varying processing technologies for reducing anti-nutritional factors and improving nutrient digestibility. However, the variations in nutritive values of CGM produced by different processing technologies are not widely known, resulting in limitation for CGM utilization in the pig diet.

In the present study, the hypothesis was that different processing technologies would affect chemical composition and nutritive value of CGM. Limited data are available describing energy contents, nutrients and amino acid (AA) digestibility in the different sources of corn processing co-products [10,11]. Therefore, the objectives of the current study were to determine the chemical composition of CGM produced using different processing technologies, and to compare the differences in the apparent total tract digestibility (ATTD) of energy and chemical composition, as well as ileal digestibility of AA, when fed to growing pigs.

## MATERIALS AND METHODS

### Animal care

All protocols used in this study were reviewed and approved by the Institutional Animal Care and Use Committee at China Agricultural University (ID: SKLAB-B-2010-003). Exp. 1 was conducted in the Metabolism Laboratory in the Swine Nutrition Research Centre of China Agricultural University (Chengde, Hebei Province, China). And Exp. 2 was conducted in the Metabolism Laboratory of China Agricultural University (Beijing, China).

### Preparation of feed ingredients

A total of 6 CGM samples processed by different processing technologies were used in the present study. Production of conventional CGM is described in detail by Archer Daniels Midland [12]. Briefly, germ is separated from other parts of the grain by steeping in a solution of bisulphate acid for about 40 h, dried, crushed, and then solvent-extracted to separate the oil from the meal. Samples of conventional CGM-1 (CGM-CV1) and -2 (CGM-CV2) were collected from different processors, which were collected from Guangyuan Grains Co. Ltd. (Shandong, China) and Zhengwang Oil Co. Ltd. (Jilin, China), respectively. Corn SL was also combined with CGM-CV1 and CGM-CV2, and the blended material was dried either with a rotary drum dryer (direct heat drying method) and a rotary steam tube dryer (indirect heat drying method), respectively. The CGM-CV1 with SL using a direct heat drying method (CGMSL-DH) was obtained from Guangyuan Grains Co. Ltd. The CGM-CV2 with SL using an indirect heat drying method (CGMSL-IH) was collected from Zhengwang Oil Co. Ltd. Corn germ expellers (CGE) were produced with no solvent extraction procedure but longer crushing time compared with CGM-CV1 and CGM-CV2, and the sample of CGE was obtained from Xingmao Co. Ltd. (Shandong, China). The dry-grind CGM (CGM-DG) was produced by a dry grinding process of “front-end fraction”. Briefly, corn is soaked in water, lightly ground in a disk attrition mill, and incubated with amylase. Germ and fiber are separated from other parts using hydrocyclones. With an aspiration step, recovered germ and pericarp fiber are separated following drying. The CGM-DG sample was obtained from Liangyou Trade Co. Ltd. (Shandong, China). Chemical composition of all tested samples was shown in Table 1.

### Animals, diets and experimental design in Exp. 1

The experiment was conducted to determine DE, ME, and ATTD of nutrients in 6 samples of CGM produced by different processing technologies using the total collection of urine and feces. Forty-two crossbred male barrows ([Landrace× Yorkshire]×Duroc) with an initial body weight (BW) of 51.2±4.5 kg and age of 95±5 d were assigned to 1 of 7 dietary treatments in a randomized complete block design with 6 replicates per dietary treatment. The pigs were placed in individual stainless steel metabolism cages (1.4 m×0.8 m×0.6 m) on concrete slatted floors and within temperature controlled-rooms (22.0±2.0°C). Cages were equipped with a feeder at the front of the cage and a nipple drinker on one side to limit feed spillage and to keep feed separate from water. In the experiment, samples of CGM were included at the expense of 30% of the energy-providing ingredients in the basal diet (Table 2). Vitamins and minerals were supplemented in all diets to meet the vitamin and mineral requirements for growing pigs recommended by NRC [13]. An analysis for chemical composition of the experimental diets is shown in Table 3. Feed was provided with an amount of 4% of pig BW and offered to pigs at 08:00 and 16:00 h in the experiment [14]. Pigs had free access to water throughout the trial. The experiment contained a 7-d adaptation to the diets and a 5-d collection period for urine and feces. The procedure for feces and urine collection refers to the previous report [15]. Briefly, feces were collected every 6 h and then immediately stored at −20°C during the collection period. A plastic bucket containing 30 mL of 6 *N* HCl was placed under each cage. Urine was collected once daily, and a 5% sub-sample was stored at −20°C.

### Animals, diets and experimental design in Exp. 2

The experiment was designed to measure the apparent ileal digestibility (AID) and standardized ileal digestibility (SID) of crude protein (CP) and AA in the different samples of CGM by using index methods [16]. Seven crossbred barrows ([Landrace×Yorkshire]×Duroc) with an initial BW of 35.3±1.3 kg were fitted with a simple T-cannula near the distal ileum and were assigned to a 7×7 Latin square design with seven periods and seven diets comprising one nitrogen-free diet and six CGM diets. Each dietary treatment included 7 replicates, and 1 pig per replicate. The diets contained a nitrogen-free diet and 6 tested diets formulated by 6 different samples of CGM with a dietary inclusion level of 40% as the sole source of dietary nitrogen (Table 2). The nitrogen-free diet was used to estimate the basal ileal endogenous losses of CP and AA. The formulation of the nitrogen-free diet was adopted from Stein et al [16]. Vitamins and minerals were supplemented in all diets to meet the vitamin and mineral requirements for growing pigs recommended by NRC [13]. The analyzed chemical composition of the experimental diets is shown in Table 4.

Feed was provided with an amount of 4% of pig BW and offered to pigs at 08:00 and 16:00 h in the experiment in each period [14]. Pigs had free access to water throughout the trial. Each period contained a 5-d adaptation to the diets and a 2-d collection period. During the collection period, digesta samples were continuously collected from 07:00 to 22:00. The procedure for ileal digesta collection refers to the previous report [16]. Briefly, a plastic bag was attached to the cannula barrel using a cable tie. Bags were replaced whenever they were filled with digesta or at least once per 30 min. Digesta was stored at −20°C immediately to prevent microbial degradation of AA.

### Sample analysis

In Exp. 1, after completing sample collection, all feces and urine were thawed and mixed thoroughly within animal and diet. A sub-sample of 40 mL urine for each pig was collected for analysis. About 350 g of feces for each pig were dried for 72 h in a 65°C drying oven and weighed. All ingredients, diets, and feces samples were ground through a 1-mm screen and analyzed for DM (method 927.05), CP (method 990.03), starch (method 979.10), ash (method 975.03), neutral (NDF) and acid detergent fiber (ADF; method 973.18) [17]. Acid-hydrolyzed ether extract (AEE) was determined by acid hydrolysis using 3 *N* HCl followed by crude fat extraction with petroleum ether using an Extraction System (Extractor, Ankom Technology, Macedon, NY, USA). Gross energy (GE) of ingredients, diets, feces and urine were determined by an Adiabatic Oxygen Bomb Calorimeter (Parr Instruments, Moline, IL, USA), with benzoic acid as the standard. Four mL of urine was added to filter paper in the bomb crucible and dried at 55°C for 24 h before determining GE [18]. Samples of ingredients, diets and feces were analyzed in duplicate, while urinary energy was analyzed in triplicate to gain an accurate result.

In Exp. 2, composition of AA were analyzed in the samples of CGM, diets and ileal digesta [17]. Before analysis, samples were hydrolyzed with 6 *N* HCl for 24 h at 110°C and analyzed for 15 AA using an Amino Acid Analyzer (L-8900; Hitachi, Tokyo, Japan). Methionine and cystine were determined as methionine sulfone and cysteic acid after cold performic acid oxidation overnight and hydrolyzing with 7.5 *N* HCl for 24 h at 110°C using an Amino Acid Analyzer (L-8800; Hitachi). Tryptophan was determined after LiOH hydrolysis for 22 h at 110°C using High Performance Liquid Chromatography (Agilent 1200 series; Agilent Technologie, Santa Clara, CA, USA). The chromium content (method 990.08) of diets and ileal digesta samples were determined after nitric acid-perchloric acid wet ash sample preparation using Polarized Zeeman Atomic Absorption Spectrometer (Z2000; Hitachi) [17].

### Calculation

The DE and ME contents and nutrient digestibility of the 6 tested CGM samples were calculated as following [19]:


$$\begin{array}{l} {\text{DE}}_{\text{d}}=[({\text{GE}}_{\text{i}}-{\text{GE}}_{\text{f}})/{\text{F}}_{\text{i}}]/0.97;\\ {\text{ME}}_{\text{d}}=[({\text{GE}}_{\text{i}}-{\text{GE}}_{\text{f}}-{\text{GE}}_{\text{u}})/{\text{F}}_{\text{i}}]/0.97;\\ {\text{DE}}_{\text{c}}=[{\text{DE}}_{\text{d}}-{\text{DE}}_{\text{b}}\times (1-0.30)]/0.30;\\ {\text{ME}}_{\text{c}}=[{\text{ME}}_{\text{d}}-{\text{ME}}_{\text{b}}\times (1-0.30)]/0.30;\\ {\text{ATTD}}_{\text{c}}=({\text{N}}_{\text{d}}\times {\text{ATTD}}_{\text{d}}-{\text{N}}_{\text{b}}\times {\text{ATTD}}_{\text{d}})/({\text{N}}_{\text{c}}\times \text{X}); \end{array}$$

The AID and SID of AA in the 6 tested CGM samples were calculated as following [20]:


$$\begin{array}{l} \text{AID}=[1-({\text{AA}}_{\text{digesta}}/{\text{AA}}_{\text{diet}})\times ({\text{Cr}}_{\text{diet}}/{\text{Cr}}_{\text{digesta}})]\times 100\%;\\ \text{IAA}={\text{AA}}_{\text{digesta}}\times ({\text{Cr}}_{\text{diet}}/{\text{Cr}}_{\text{digesta}});\\ \text{SID}=\text{AID}+(\text{IAA}/{\text{AA}}_{\text{ingredient}})\times 100\%; \end{array}$$

where GE_i_ is the total GE intake of each pig (kcal of DM), and F_i_ which is the actual feed intake over the 5-d collection period; GE_f_ and GE_u_ are the GE content in feces and urine of each pig (kcal of DM) over the 5-d collection period; DE_d_ and ME_d_ are the DE and ME values in each diet (kcal/kg of DM) and 0.97 is the percentage of corn and soybean meal in the basal diet; DE_c_ and ME_c_ are the DE and ME values in tested CGM (kcal/kg of DM), and 0.30 is the percentage of tested CGM in the diets; the DE_b_ and ME_b_ are the DE and ME values in the basal diet (kcal/kg of DM); ATTD_d_ (%) and ATTD_c_ (%) are total tract digestibility of nutrients in the diet and test CGM; N_d_, N_b_ and N_c_ are nutrients contents in the tested diet, basal diet and tested CGM; X is a proportion of nutrients in tested CGM accounted for the tested diets; AA_digesta_ and Cr_digesta_ are the contents of AA and chromium in the ileal digesta (g/kg of DM), while AA_diet_ and Cr_diet_ are the contents of AA and chromium in the test diets (g/kg of DM); The basal ileal endogenous loss of each AA (IAA, g/kg of DM intake) was determined for pigs fed the N-free diet; AA_ingredient_ is the contents of AA in the CGM samples (g/kg of DM); AA_ingredient_ is AA contents in the tested CGM samples.

### Statistical analysis

Data on the ATTD of nutrients, DE and ME contents were analyzed by generalized linear model using the Mixed Procedure (Verseion 9.4; SAS Institute, Cary, NC, USA) as a randomized complete block design. The fixed effect was the 6 CGM sources, and block was considered as random effects. Data on the AID and SID of AA were analyzed using the MIXED procedure (Verseion 9.4; SAS Institute). An ANOVA was conducted, with diet as the main effect and pig and period as random effects. The pig was considered as the experimental unit. The LSMEANS procedure was used to calculate treatment means, and statistical differences among different samples of CGM were performed by Tukey’s multiple range test. The differences were considered as significant when p<0.05 as a tendency when 0.05≤p<0.10.

## RESULTS

There was no residual feed left by any pig during the whole experiment. All pigs were healthy with no clinical symptoms and cannula losses.

### Composition of energy and chemical constituents in 6 corn germ meal samples

Analyzed energy and chemical composition of CGM produced by different processing technologies are shown in Table 1. The results showed contents of GE, CP, AEE, starch, NDF, ADF, Ca and P in the 6 tested CGM samples ranged from 16.3 MJ/kg to 19.3 MJ/kg, from 17.2% to 28.6%, from 1.8% to 7.1%, from 10.5% to 28.6%, from 27.2% to 49.7%, from 5.6% to 14.5%, from 0.02% to 0.45%, and from 0.31% to 1.54%, respectively. Contents of lysine (Lys), methionine (Met), tryptophan (Trp), threonine (Thr) and valine (Val) in the 6 tested CGM samples ranged from 0.66% to 1.04%, from 0.30% to 0.47%, from 0.09% to 0.22%, from 0.70% to 1.04%, and from 0.99% to 1.52%, respectively.

### Energy contents and apparent total tract digestibility of chemical constituents

Results for the differences in nutrient digestibility and energy contents among different CGM samples are shown in Table 5. There were differences in the ATTD of GE and nutrients except for NDF (p = 0.07), and energy contents (as-fed basis) among 6 samples of CGM (p<0.05). The ATTD of ADF and GE in CGMSL-IH were greater (p<0.05) than those in CGM-CV2 for pigs. The ATTD of organic matter (OM), CP and GE in CGMSL-DH were greater (p<0.05) than those in CGM-CV1 for pigs. The ATTD of GE and AEE, and contents of DE and ME (as-fed basis) in CGE were greater (p<0.05) than those in CGM-CV1. The ATTD of OM and GE in CGM-DG were greater (p<0.05) than those in CGM-CV1. In addition, compared with the CGM-CV1, CGM-CV2 showed a greater (p<0.05) ATTD of GE for growing pigs.

### Amino acid digestibility

Results for the differences in the AID and SID of CP and AA among different CGM samples are shown in Tables 6, 7. There were differences in the AID and SID of CP and AA among 6 samples of CGM (p<0.05). The AID and SID of CP and AA, except for Trp and cysteine (Cys), in CGMSL-IH were greater (p<0.05) than those in CGM-CV2. The AID and SID of CP and AA, except for alanine (Arg), histidine (His), Lys and tyrosine (Tyr), in CGMSL-DH were greater (p<0.05) than those in CGM-CV1. The AID and SID of CP and most AA, except for the Arg, His, Lys, phenylalanine (Phe), Cys, glycine and Tyr, in CGE were greater (p<0.05) than those in CGM-CV1. The AID and SID of CP and all AA in CGM-DG were greater (p<0.05) than those in CGM-CV1. In addition, compared with the CGM-CV1, CGM-CV2 showed a greater (p<0.05) AID and SID of CP, Met, Thr, Val and alanine (Ala) for growing pigs.

## DISCUSSION

### Chemical composition of corn germ meal

Contents of DM, AEE, ash, starch, and GE in CGM-CV1 and CGM-CV2 agreed with those values reported by NRC [13] and Rojas et al [10]. Contents of CP and all AA, except for Leucine, Phe, Val, and Glu, in CGM-CV1 were less than those shown in NRC [13] and Rojas et al [10], and the content of NDF in CGM-CV2 was less than reported values in NRC [13]. Contents of DM, CP, AEE, NDF, and ADF in CGMSL-DH and CGMSL-IH agreed with those values reported by Liu et al [9]. However, GE contents in all tested ingredients were slightly lower than values reported by Shi et al [9], mainly due to a lower starch content. Contents of CP and ash in CGMSL-DH and CGMSL-IH were greater than CGM-CV1 and CGM-CV2 in the present study. This observation mentioned above is likely due to high contents of ash and water-soluble proteins in the steep water solids [21]. All AA contents in CGMSL-DH and CGMSL-IH were greater than CGM-CV1 and CGM-CV2, respectively, except for Lys. No difference in Lys content between CGM-CV1 and CGMSL-DH indicates that heat damage may occur during direct heating process and may impair Lys content in the feed ingredients [22].

Compared with the values published in the previous report [6], greater CP and fiber, but less AEE and starch contents, lead to similar GE in CGE used in the present study. The CGM-DG used in this experiment was produced from dry-grind processor and contained more starch and ash than CGM produced from the wet-milling processor [9,13]. A small amount of endosperm, which contain the major portion of the starch and protein, remain attached to the germ and therefore increased the starch and protein of the byproduct and decreased the fiber in the ingredient. The greater ash content in the CGM-DG is likely caused by the corn cob inclusion in the dry-grind process.

### The apparent total tract digestibility of chemical constituents

In the present study, CGMSL-DH and CGM-CV1 were sourced from the same processing plant and the same batch of CGM-CV1 was used to produce CGMSL-DH, as well as CGMSL-IH and CGM-CV2. In the two comparisons (CGM-CV1 vs. CGMSL-DH and CGM-CV2 vs. CGMSL-IH), the increment of CP (approximately 11 and 5 percentage units, respectively) and ATTD of CP (approximately 16% and 8%, respectively) are both different. The greater content and digestibility of CP in CGMSL-DH compared with CGM-CV1 indicates that adding SL to CGM contributes to more digestible protein. These differences are likely due to different sources of SL and the ratio of SL to CGM used in producing CGMSL in the different processors. In addition, the different contributions to the increased ATTD of CP between CGM-CV1 and CGM-CV2 by adding SL could be associated with their varying chemical composition and the SID of some AA. The greater ATTD of OM and GE in CGM-DG than in CGM-CV1 is likely due to the lower content of fiber components. Similarly, the CGM-CV2 showed greater ATTD of GE compared with the CGM-CV1 due to the lower contents of NDF and ADF. Consistent with the previous studies [23,24], varying contents of NDF and ADF between CGM-CV1 (Shandong province, China; 36 degrees north of the Equator) and CGM-CV2 (Jilin province, China; 45 degrees north of the Equator) derived from the different variety and planting environment of the corn.

The ATTD of AEE in all kinds of CGM samples, except for CGE, was below 50%. A possible reason for this observation is that the endogenous losses of fat contribute relatively more to fat output when ether extract (EE) content in the diet is low, which results in low calculated value for oil digestibility. This fact is especially true in low EE and high-fiber feed ingredients [25,26]. Therefore, the content of endogenous losses of AEE to the total output was less in pigs fed a CGE diet compared with the pigs fed other diets, leading to greater ATTD of AEE digestibility in CGE. The EE in high-fiber ingredients, especially in corn processing co-products, is thought to be encapsulated in cell membranes or fiber compounds and is assumed hard to be digested, which is likely another reason for the low digestibility in CGM-CV1, CGMSL-DH and CGM-DG. The CGE was produced with no solvent extraction and longer crushing time compared with the other test feed ingredients [6]. It is possible that the crushing process contributes to the increased oil digestibility. Kim et al [26] reported that heating could reduce digestibility of AEE in corn processing co-products. However, the fact that the ATTD of AEE in CGE agrees with the value of extracted corn oil indicates that heating during corn processing co-products production may not impair EE digestibility of these feed ingredients [22]. On the contrary, heating, as well as crushing, during CGM production can help to increase EE digestibility. This observation mentioned above shows a possibility to replace, at least part of, extracted corn oil or soybean oil with CGE in the pig diets. In addition, the CGM-CV2 showed a lower ATTD of AEE compared with the CGM-CV1, which may be associated with more endogenous loss of fat in the CGM-CV2 group, but the hypothesis should be further evaluated.

Surprisingly, the ATTD of ADF in CGMSL-IH were greater than in CGM-CV2, whereas the ATTD of ADF in CGMSL-DH were not different compared with the CGM-CV1. The increased digestibility of fiber in CGMSL-IH compared with CGM-CV2 is not evident, indicating that inclusion of SL could not affect digestibility of NDF and ADF [9]. In addition, greater fiber digestibility in CGMSL-IH implies that complex fiber compounds may be changed to smaller molecules, which is associated with greater ATTD of CP and AEE. However, no differences in the ATTD of CP and AEE were observed between CGMSL-IH and CGM-CV2. The ATTD of NDF and ADF in CGM samples used in the present study were greater than those reported value in corn dis-tillers dried grains with solubles [27], corn bran [28], corn fiber [29], or corn bran with solubles [30]. The greater fiber digestibility in CGM samples used in our study may be caused by varying fiber structure between corn germ and the other parts of corn, as well as the physical and chemical processes used in the dry-grind and wet-milling processing plants. Processing technologies of grinding and heating could modify the structure of fiber components and increase the surface of the enzymatic digestion reaction, which might make fiber components more digestible than raw feed ingredients [31].

### Energy contents and the apparent total tract digestibility of gross energy

The ATTD of GE and contents of DE and ME in CGM-CV1 and CGM-CV2 obtained in this study were less than those values published by Rojas et al [10] and NRC [13], which is likely a result of a relatively low content of CP in CGM-CV1 and CGM-CV2 used in this study, but greater than those values reported by Gutierrez et al [30]. The CGM-CV2 showed a greater ATTD of GE compared with the CGM-CV1 due to the lower contents of NDF and ADF. The ATTD of GE and contents of DE and ME in CGMSL-DH and CGMSL-IH were less than those reported values [9], which is likely due to a relatively low starch content in CGMSL-DH and CGMSL-IH. However, the ATTD of GE in CGMSL-DH and CGMSL-IH agree with the value reported for CGM [13], and the DE and ME values for CGMSL-DH and CGMSL-IH agree with the values reported for CGM [10]. The DE and ME values for CGE obtained in this experiment were less than those reported values [6]. The decreased content of digestible AEE, as well as the increased contents of NDF and ADF, is likely the reason for the reduced contents of DE and ME in the tested CGM. The increased digestible AEE content and low fiber content lead to greater contents of DE and ME in CGE than those in CGM-CV1 in the present study. No publications on DE and ME in CGM-DG were found, and the increased starch content in CGM-DG compared with CGM-CV1 should play a crucial role in the greater ATTD of GE in CGM-DG than in CGM-CV1, because starch is often considered to be highly digestible as a primary energy source [32]. In addition, the greater ATTD of CP in CGM-DG compared with CGM-CV1 is another possible reason for the greater energy digestibility.

### Amino acids digestibility

The ileal AA digestibility is affected by the endogenous loss of dietary nitrogen [33]. The endogenous loss of nitrogen is comprised of two components, namely basal and specific losses. The basal losses are fixed and associated with feed dry matter intake, whereas the specific losses are variable and induced by the presence of dietary components, such as fibre and anti-nutrients. Therefore, it is necessary to determine the SID of AA by using AID of AA to correct the endogenous loss of AA. A nitrogen-free diet was used to determine the basal endogenous loss of AA in the present study. The AID and SID of CP and AA in CGM-CV1 and CGM-CV2 were less than those values reported by NRC [13]. This observation indicates that there were large variations on CP and AA digestibility of 6 CGM samples produced among different countries for growing pigs due to the different varieties, planting area and processing technologies [34]. However, there is a limitation on studying effects of genetic cultivars and growth environment on AA digestibility for CGM samples used in the present study. In the present study, the CGM-CV2 showed greater AID and SID of CP, Met, Thr, Val and Ala compared with the CGM-CV1 due to the lower contents of fiber components. The values for AID and SID of CP and AA in CGMSL-DH and CGMSL-IH that were determined in the present study are greater than previous data [9], although the CP and AA contents in the publications above are very close. Specifically, the value of the AID of Lys in CGMSL-IH is greater compared with the reported value [9]. However, the values for the AID and SID of CP and AA in CGMSL-IH and CGMSL-DH used in the present study concur with previously published values in CGM from North America [13] and Europe [6]. The much greater AID and SID of CP and most AA in CGMSL-DH and CGMSL-IH than in CGM-CV1 and CGM-CV2 are likely a result of adding corn SL into CGM-CV. The SL is produced by steeping corn grain in a solution of bisulphate acid for about 40 h, and therefore mainly contains disassociative AA and organic acids, which is assumed much more digestible than the protein remaining in the corn kernel. Another reason is that the CGMSL-DH and CGMSL-IH contain lower contents of NDF and ADF than CGM-CV1 and CGM-CV2. The fact that the AID and SID of Lys in CGMSL-IH were greater than in CGM-CV2, whereas the AID and SID of Lys in CGMSL-DH did not differ from the values in CGM-CV1, indicating that heat damage may impair Lys digestibility to a greater extent during direct heat compared with indirect heat [35].

The values for the SID of AA in CGE determined in Europe have been reported [6], and the SID of AA determined in the present study are less than European values. The main reason for this observation is that the CGE used in this experiment contains greater contents of NDF and ADF, which have an impaired influence on the values for AA digestibility. The greater digestibility of most AA in CGE compared with CGM-CV1 indicated the potential to applying into the pig diets. The reason for the greater CP and AA digestibility in CGM-DG than in CGM-CV1 could be caused by the lower contents of fiber components. This observation concurs with the greater AA digestibility in corn gluten meal [36], mainly obtained from endosperm.

## CONCLUSION

There are large variations on DE, ME, energy and AA digestibility among conventional CGM, CGMSL-DH, CGMSL-IH, CGE and CGM-DG. CGMSL-IH and CGM-DG are recommended to be used in pig production considering the high AA digestibility, and the CGE is also recommended due to the high available energy content. Furthermore, the effects of genetic and environmental variability of the corn for producing CGM used in this study should be further explored.

## Figures and Tables

**Table 1:** Analyzed energy and chemical composition of corn germ meals produced using different processing methods (as-fed basis, %)

Item	CGM-CV1	CGM-CV2	CGMSL-DH	CGMSL-IH	CGE	CGM-DG
Dry matter	90.9	93.1	91.4	91.1	93.5	91.4
Crude protein	17.2	22.7	28.6	27.2	21.7	19.5
Acid-hydrolyzed ether extract	3.4	2.0	1.8	2.1	7.1	2.0
Neutral detergent fiber	49.7	37.9	27.2	30.8	45.5	28.8
Acid detergent fiber	14.5	10.1	7.7	8.4	13.2	5.6
Ash	2.6	5.0	8.1	6.7	2.8	7.4
Calcium	0.07	0.10	0.45	0.04	0.05	0.02
Phosphorus	0.31	0.71	0.52	1.23	0.48	1.54
Starch	15.4	12.4	10.5	11.1	13.0	28.6
Gross energy (MJ/kg)	17.7	17.6	16.9	17.2	19.3	16.3
Indispensable AA						
Arg	0.95	1.13	0.75	1.43	1.13	1.37
His	0.60	0.77	0.73	0.86	0.67	0.59
Ile	0.58	0.75	0.85	0.88	0.71	0.60
Leu	1.53	1.99	2.43	2.37	1.77	1.52
Lys	0.73	0.84	0.66	1.04	0.79	0.94
Met	0.30	0.36	0.44	0.47	0.40	0.30
Phe	0.85	0.96	1.04	1.10	0.96	0.90
Thr	0.70	0.89	1.00	1.04	0.83	0.74
Trp	0.13	0.16	0.09	0.15	0.16	0.22
Val	0.99	1.29	1.50	1.52	1.19	1.01
Dispensable AA						
Ala	1.23	1.82	2.36	2.26	1.49	1.42
Asp	1.12	1.36	1.42	1.69	1.37	1.48
Cys	0.35	0.48	0.56	0.50	0.40	0.34
Glu	2.28	3.04	3.49	3.56	2.70	2.48
Gly	0.90	1.14	1.32	1.34	1.09	1.01
Pro	1.10	1.73	2.17	2.03	1.22	0.95
Ser	0.76	0.92	1.01	1.13	0.91	0.87
Tyr	0.35	0.46	0.33	0.56	0.41	0.39

CGM-CV, conventional corn germ meal; CGMSL-DH, corn germ meal with steep liquor applied using direct heat; CGMSL-IH, corn germ meal with steep liquor applied using indirect heat; CGE, corn germ expellers; CGM-DG, dry-grind corn germ meal; AA, amino acid; Arg, arginine; His, histidine; Ile, isoleucine; Leu, leucine; Lys, lysine; Met, methionine; Phe, phenylalanine; Thr, threonine; Trp, tryptophane; Val, valine; Ala, alanine; Asp, aspartic acid; Cys, cysteine; Glu, glutamic acid; Gly, glycine; Pro, proline; Ser, serin; Tyr, tyrosine.

**Table 2:** Ingredient composition of the experimental diets (as-fed basis, %)

Item	Exp. 1		Exp. 2	
	
	Basal diet	Test diets	Corn germ meal diets	N-free diet
Corn	76.00	53.20	-	-
Soybean meal (46% crude protein)	21.00	14.70	-	-
Corn germ meal	-	29.10	40.00	-
Corn starch	-	-	40.00	73.35
Soybean oil	-	-	2.00	3.00
Sucrose	-	-	15.00	15.00
Cellulose^1)^	-	-	-	4.00
Dicalcium phosphate (18.5% phosphorus)	1.10	1.10	1.50	2.50
Limestone	0.90	0.90	0.40	0.50
Sodium chloride	0.30	0.30	0.30	0.45
Choline chloride	0.20	0.20	-	-
Chromic oxide	-	-	0.30	0.30
Potassium carbonate	-	-	-	0.30
Magnesium oxide	-	-	-	0.10
Mineral and vitamin premix^2)^	0.50	0.50	0.50	0.50

1) Made by Chemical Reagents Company (Beijing, China).

2) Provided the following quantities of vitamins and minerals per kg of complete diet: vitamin A, 5,512 IU; vitamin D3, 2,200 IU; vitamin E, 30 IU; vitamin K_3_, 2.2 mg; vitamin B_12_, 27.6 μg; riboflavin, 4 mg; pantothenic acid, 14 mg; niacin, 30 mg; choline chloride, 400 mg; folacin, 0.7 mg; thiamine 1.5 mg; pyridoxine 3 mg; biotin, 44 μg; Mn, 40 mg (MnO); Fe, 75 mg (FeSO_4_·H_2_O); Zn, 75 mg (ZnO); Cu, 100 mg (CuSO_4_·5H_2_O); I, 0.3 mg (KI); Se, 0.3 mg (Na_2_SeO_3_).

**Table 3:** Chemical composition of experimental diets used in Exp. 1 (as-fed basis, %)

Item	Basal diet	Test diets					

		CGM-CV1	CGM-CV2	CGMSL-DH	CGMSL-IH	CGE	CGM-DG
DM	87.4	87.8	88.3	88.0	88.8	89.2	89.6
CP	15.6	16.3	17.4	20.2	19.9	18.0	17.7
AEE	3.0	3.1	2.7	2.6	2.7	4.2	2.7
NDF	11.2	24.1	22.6	20.4	20.5	24.9	19.9
ADF	3.7	8.5	7.3	6.3	6.3	6.8	5.6
Ash	4.3	4.5	5.0	6.0	5.6	4.6	5.8
GE (MJ/kg)	16.1	16.5	16.4	16.1	16.0	16.7	16.0

CGM-CV, conventional corn germ meal; CGMSL-DH, corn germ meal with steep liquor applied using direct heat; CGMSL-IH, corn germ meal with steep liquor applied using indirect heat; CGE, corn germ expellers; CGM-DG, dry-grind corn germ meal; DM, dry matter; CP, crude protein; AEE, acid hydrolyzed ether extract; NDF, neutral detergent fiber; ADF, acid detergent fiber; GE, gross energy.

**Table 4:** Chemical composition of experimental diets used in Exp. 2 (as-fed basis, %)

Item	Test diet						N-free diet

	CGM-CV1	CGM-CV2	CGMSL-DH	CGMSL-IH	CGE	CGM-DG	
CP	7.38	9.72	11.60	11.47	8.71	7.79	0.37
Indispensable AA							
Arg	0.39	0.38	0.32	0.58	0.45	0.51	0.01
His	0.25	0.28	0.36	0.39	0.29	0.26	0.01
Ile	0.24	0.25	0.38	0.36	0.26	0.22	0.01
Leu	0.69	0.73	1.21	1.07	0.75	0.65	0.05
Lys	0.30	0.29	0.27	0.44	0.33	0.39	0.00
Met	0.11	0.13	0.16	0.15	0.12	0.11	0.00
Phe	0.31	0.28	0.40	0.40	0.33	0.28	0.02
Thr	0.28	0.31	0.44	0.43	0.33	0.29	0.01
Trp	0.07	0.06	0.04	0.04	0.05	0.09	0.00
Val	0.48	0.50	0.81	0.72	0.53	0.44	0.01
Dispensable AA							
Ala	0.52	0.64	1.07	0.98	0.64	0.59	0.02
Asp	0.48	0.50	0.66	0.74	0.59	0.62	0.02
Cys	0.19	0.24	0.30	0.24	0.20	0.22	0.12
Glu	1.13	1.28	1.86	1.81	1.34	1.20	0.04
Gly	0.33	0.32	0.52	0.48	0.36	0.33	0.01
Pro	0.43	0.58	0.96	0.88	0.47	0.36	0.03
Ser	0.31	0.32	0.44	0.47	0.37	0.35	0.02
Tyr	0.17	0.19	0.16	0.23	0.20	0.19	0.02

CGM-CV, conventional corn germ meal; CGMSL-DH, corn germ meal with steep liquor applied using direct heat; CGMSL-IH, corn germ meal with steep liquor applied using indirect heat; CGE, corn germ expellers; CGM-DG, dry-grind corn germ meal; CP, crude protein; AA, amino acid; Arg, arginine; His, histidine; Ile, isoleucine; Leu, leucine; Lys, lysine; Met, methionine; Phe, phenylalanine; Thr, threonine; Trp, tryptophane; Val, valine; Ala, alanine; Asp, aspartic acid; Cys, cysteine; Glu, glutamic acid; Gly, glycine; Pro, proline; Ser, serin; Tyr, tyrosine.

**Table 5:** Apparent total tract digestibility of nutrients and energy contents in the different samples of corn germ meal for growing pigs

Items	CGM-CV1	CGM-CV2	CGMSL-DH	CGMSL-IH	CGE	CGM-DG	SEM^1)^	p-value^2)^
Apparent total tract digestibility (%)								
DM	65.9^a^	69.5^ab^	72.4^ab^	74.7^b^	68.8^ab^	71.8^ab^	1.90	0.001
OM	65.3^a^	71.0^ab^	72.7^b^	76.6^b^	69.9^ab^	75.5^b^	1.97	0.001
CP	73.6^a^	78.0^ab^	89.7^c^	85.6^bc^	80.3^ab^	81.7^ab^	2.19	0.005
AEE	48.3^b^	22.4^a^	38.8^ab^	44.4^ab^	85.6^c^	37.0^ab^	5.94	0.014
NDF	64.7	67.2	65.4	74.3	66.8	68.4	2.27	0.072
ADF	60.2^a^	64.8^a^	60.1^a^	72.7^b^	58.9^a^	65.3^a^	2.20	0.011
GE	66.0^a^	69.0^b^	71.3^bc^	72.5^c^	68.6^b^	75.2^c^	0.61	0.005
As-fed basis (MJ/kg)								
DE	11.69^a^	12.14^ab^	12.06^ab^	12.44^ab^	13.22^b^	12.24^ab^	0.34	0.017
ME	11.08^a^	11.36^ab^	11.63^ab^	11.79^ab^	12.48^b^	11.80^ab^	0.32	0.008
DM basis (MJ/kg)								
DE	12.86	13.04	13.20	13.65	14.14	13.39	0.37	0.069
ME	12.19	12.20	12.74	12.94	13.34	12.90	0.36	0.062

1) Pooled standard error of the LSMeans.

a–c Superscripts of the mean within a row with different letters indicate a significant difference among different dietary treatments (p<0.05), n = 6.

CGM-CV, conventional corn germ meal; CGMSL-DH, corn germ meal with steep liquor through direct heat; CGMSL-IH, corn germ meal with steep liquor through indirect heat; CGE, corn germ expellers; CGM-DG, dry-grind corn germ meal; DM, dry matter; OM, organic matter; CP, crude protein; AEE, acid hydrolyzed ether extract; NDF, neutral detergent fiber; ADF, acid detergent fiber; GE, gross energy; DE, digestible energy; ME, metabolizable energy.

**Table 6:** Apparent ileal digestibility (%) of CP and AA in the different samples of corn germ meal for growing pigs

Items	CGM-CV1	CGM-CV2	CGMSL-DH	CGMSL-IH	CGE	CGM-DG	SEM^1)^	p-value^2)^
CP	22.7^a^	43.3^b^	57.0^c^	61.9^c^	44.0^b^	54.4^bc^	3.28	0.001
Indispensable AA								
Arg	66.9^a^	65.3^a^	68.4^a^	81.9^b^	75.2^ab^	81.5^b^	2.66	0.001
His	67.7^a^	68.7^a^	78.0^b^	80.8^b^	72.4^ab^	78.5^b^	2.26	0.001
Ile	46.4^a^	52.4^b^	70.6^d^	71.3^d^	57.8^bc^	64.3^cd^	2.49	0.001
Leu	60.9^a^	67.5^b^	81.2^d^	80.3^d^	69.1^b^	75.1^c^	1.72	0.001
Lys	56.4^a^	57.7^a^	53.9^a^	74.1^c^	62.8^ab^	70.2^bc^	2.9	0.001
Met	64.3^a^	71.7^b^	79.9^c^	78.7^c^	71.9^b^	77.9^bc^	2.07	0.001
Phe	60.5^a^	62.1^a^	74.8^b^	76.7^b^	66.8^a^	73.2^b^	2.07	0.001
Thr	23.0^a^	38.0^b^	56.6^cd^	61.0^d^	44.0^bc^	55.8^cd^	4.02	0.001
Trp	34.1^c^	35.4^c^	0.5^d^	22.4^bc^	12.1^cd^	61.4^d^	5.00	0.001
Val	56.1^a^	62.0^b^	77.7^c^	76.2^c^	64.4^b^	71.0^c^	1.96	0.001
Dispensable AA								
Ala	48.5^a^	59.6^b^	79.1^d^	78.0^d^	64.1^bc^	69.4^c^	2.38	0.001
Asp	31.7^a^	36.2^a^	57.5^bc^	63.6^c^	49.7^b^	65.6^c^	3.91	0.001
Cys	32.4^a^	47.3^b^	60.9^c^	55.7^bc^	43.9^b^	63.8^c^	3.59	0.001
Glu	57.0^a^	58.8^a^	74.2^b^	75.8^b^	69.9^b^	77.0^b^	2.64	0.001
Gly	−28.6^a^	−31.8^a^	40.3^b^	30.3^b^	5.0^ab^	26.5^b^	10.92	0.001
Pro	−59.8^a^	−16.5^ab^	67.6^c^	59.1^c^	3.1^b^	28.0^bc^	15.85	0.001
Ser	37.6^a^	46.9^ab^	62.4^cd^	68.2^d^	55.2^bc^	65.2^cd^	3.41	0.001
Tyr	52.2^a^	54.2^a^	51.6^a^	71.0^b^	61.6^ab^	70.9^b^	3.57	0.001

1) Pooled standard error of the LSMeans.

a–d Superscripts of the mean within a row with different letters indicate a significant difference among different dietary treatments (p<0.05), n = 6.

CP, crude protein; AA, amino acids; CGM-CV, conventional corn germ meal; CGMSL-DH, corn germ meal with steep liquor through direct heat; CGMSL-IH, corn germ meal with steep liquor through indirect heat; CGE, corn germ expellers; CGM-DG, dry-grind corn germ meal; Arg, arginine; His, histidine; Ile, isoleucine; Leu, leucine; Lys, lysine; Met, methionine; Phe, phenylalanine; Thr, threonine; Trp, tryptophane; Val, valine; Ala, alanine; Asp, aspartic acid; Cys, cysteine; Glu, glutamic acid; Gly, glycine; Pro, proline; Ser, serin; Tyr, tyrosine.

**Table 7:** Standardized ileal digestibility (%) of CP and AA in the different samples of corn germ meal for growing pigs

Items^1)^	CGM-CV1	CGM-CV2	CGMSL-DH	CGMSL-IH	CGE	CGM-DG	SEM^2)^	p-value^3)^
CP	40.6^a^	56.9^b^	68.3^cd^	73.4^d^	59.2^bc^	71.3^d^	3.28	0.001
Indispensable AA								
Arg	75.2^a^	73.7^a^	78.4^ab^	87.4^b^	82.4^ab^	87.8^b^	2.66	0.001
His	74.3^a^	74.6^a^	82.7^ab^	85.1^b^	78.2^ab^	84.9^b^	2.26	0.001
Ile	56.5^a^	62.2^ab^	77.0^c^	78.1^c^	67.0^b^	75.2^c^	2.49	0.001
Leu	68.2^a^	74.3^ab^	85.3^c^	85.0^c^	75.8^b^	82.7^c^	1.72	0.001
Lys	62.5^a^	64.0^a^	60.9^a^	78.3^b^	68.5^ab^	75.0^b^	2.90	0.001
Met	69.3^a^	76.7^b^	84.9^c^	83.7^c^	76.9^b^	82.9^bc^	2.07	0.001
Phe	69.6^a^	72.1^a^	81.9^b^	83.8^b^	75.5^a^	83.1^b^	2.07	0.001
Thr	40.8^a^	54.5^b^	68.1^bc^	72.7^c^	59.3^bc^	73.0^c^	4.02	0.001
Trp	46.7^bc^	50.2^c^	22.5^a^	44.5^bc^	29.6^ab^	71.0^d^	5.11	0.001
Val	61.9^a^	67.6^b^	81.1^c^	80.1^c^	69.6^b^	77.3^c^	1.96	0.001
Dispensable AA								
Ala	59.9^a^	68.9^b^	84.6^d^	84.0^d^	73.3^bc^	79.4^cd^	2.38	0.001
Asp	43.8^a^	47.9^a^	66.2^bc^	71.5^bc^	59.5^b^	75.0^c^	3.91	0.001
Cys	54.9^a^	64.9^ab^	75.6^bc^	74.0^bc^	65.8^ab^	83.1^c^	3.59	0.001
Glu	63.6^a^	64.7^a^	78.2^b^	79.9^b^	75.5^ab^	83.1^b^	2.64	0.001
Gly	18.9^a^	15.9^a^	69.8^b^	62.8^b^	47.4^ab^	73.3^b^	10.92	0.001
Pro	18.6^a^	42.0^ab^	103.0^c^	97.4^c^	75.7^bc^	122.1^c^	15.85	0.001
Ser	52.1^a^	60.7^ab^	72.5^c^	77.7^c^	67.1^bc^	77.9^c^	3.41	0.001
Tyr	68.9^a^	69.3^a^	69.3^a^	83.2^b^	75.6^ab^	85.4^b^	3.57	0.001

1) The basal ileal endogenous loss of CP and each AA (g/kg of DM intake) was determined for pigs fed the nitrogen-free diet, and the SID of AA was calculated by using the AID of AA to correct endogenous nitrogen loss. The basal ileal endogenous loss of CP, Arg, His, Ile, Leu, Lys, Met, Phe, Thr, Trp, Val, Ala, Asp, Cys, Glu, Gly, Pro, Ser, Tyr were 33.87, 0.87, 0.44, 0.64, 1.23, 0.49, 0.17, 0.85, 1.37, 0.18, 0.63, 1.54, 1.49, 0.87, 1.66, 4.70, 9.49, 1.21 and 0.64 g/kg of DM intake.

2) Pooled standard error of the LSMeans.

a–d Superscripts of the mean within a row with different letters indicate a significant difference among different dietary treatments (p<0.05), n = 6.

CP, crude protein; AA, amino acids; CGM-CV, conventional corn germ meal; CGMSL-DH, corn germ meal with steep liquor through direct heat; CGMSL-IH, corn germ meal with steep liquor through indirect heat; CGE, corn germ expellers; CGM-DG, dry-grind corn germ meal; Arg, arginine; His, histidine; Ile, isoleucine; Leu, leucine; Lys, lysine; Met, methionine; Phe, phenylalanine; Thr, threonine; Trp, tryptophane; Val, valine; Ala, alanine; Asp, aspartic acid; Cys, cysteine; Glu, glutamic acid; Gly, glycine; Pro, proline; Ser, serin; Tyr, tyrosine.
